# Automated scoring of collaterals, blood pressure, and clinical outcome after endovascular treatment in patients with acute ischemic stroke and large-vessel occlusion

**DOI:** 10.3389/fneur.2022.944779

**Published:** 2022-08-09

**Authors:** Daniel Guisado-Alonso, Pol Camps-Renom, Raquel Delgado-Mederos, Esther Granell, Luis Prats-Sánchez, Alejandro Martínez-Domeño, Marina Guasch-Jiménez, M. Victoria Acosta, Anna Ramos-Pachón, Joan Martí-Fàbregas

**Affiliations:** ^1^Stroke Unit, Department of Neurology, Hospital de la Santa Creu i Sant Pau, Biomedical Research Institute Sant Pau, Universitat Autònoma de Barcelona (Department of Medicine), Barcelona, Spain; ^2^Department of Radiology, UDIAT Corporació Sanitària Parc Taulí, Sabadell, Spain

**Keywords:** collateral circulation, acute stroke, endovascular treatment, blood pressure, outcome

## Abstract

**Introduction:**

We aimed to determine whether the degree of collateral circulation is associated with blood pressure at admission in acute ischemic stroke patients treated with endovascular treatment and to determine its prognostic value.

**Methods:**

We evaluated patients with anterior large vessel occlusion treated with endovascular treatment in a single-center prospective registry. We collected clinical and radiological data. Automated and validated software (Brainomix Ltd., Oxford, UK) was used to generate the collateral score (CS) from the baseline single-phase CT angiography: 0, filling of ≤10% of the occluded MCA territory; 1, 11–50%; 2, 51–90%; 3, >90%. When dichotomized, we considered that CS was good (CS = 2–3), or poor (CS = 0–1). We performed bivariate and multivariable ordinal logistic regression analysis to predict CS categories in our population. The secondary outcome was to determine the influence of automated CS on functional outcome at 3 months. We defined favorable functional outcomes as mRS 0–2 at 3 months.

**Results:**

We included 101 patients with a mean age of 72.1 ± 13.1 years and 57 (56.4%) of them were women. We classified patients into 4 groups according to the CS: 7 patients (6.9%) as CS = 0, 15 (14.9%) as CS = 1, 43 (42.6%) as CS = 2 and 36 (35.6%) as CS = 3. Admission systolic blood pressure [aOR per 10 mmHg increase 0.79 (95% CI 0.68–0.92)] and higher baseline NIHSS [aOR 0.90 (95% CI, 0.84–0.96)] were associated with a worse CS. The OR of improving 1 point on the 3-month mRS was 1.63 (95% CI, 1.10–2.44) favoring a better CS (*p* = 0.016).

**Conclusion:**

In acute ischemic stroke patients with anterior large vessel occlusion treated with endovascular treatment, admission systolic blood pressure was inversely associated with the automated scoring of CS on baseline CT angiography. Moreover, a good CS was associated with a favorable outcome.

## Introduction

Endovascular treatment (EVT) is the standard of care for acute ischemic stroke (AIS) in selected patients with large intracranial vessel occlusion (LVO) (1, 2). However, only around 46% of the patients treated with EVT achieve functional independence at 3 months (3). Therefore, there is still room for improvement in EVT clinical outcomes.

Pre-treatment degree of collateral circulation (CC) has been reported as an important determinant for successful reperfusion (4) and clinical outcome (3) after EVT. The effects of the CC are crucial in maintaining perfusion to penumbral regions and also in facilitating the clearance of fragmented thrombus (5). In some studies, a higher admission blood pressure (BP) (6) and BP drops during EVT (7) are associated with a poorer clinical outcome after EVT. However, there are scarce and contradictory data evaluating the effect of admission BP on CC in AIS (8, 9). A better understanding of this relationship could lead to an optimisation of CC by a better management of pre-procedural and intra-procedural BP.

Multiple scores are available to measure CC in AIS, but the intra- and inter-observer agreement for all of them is modest (10, 11). Automated quantitative CC scoring in patients with AIS is a reliable, quick, and user-independent measure of the CC degree on baseline Computed Tomography Angiography (CTA) (12). Although there is no gold standard, one of the most widely used CC scales on CTA is the one described by Tan et al. (13). For this scale, there is a validated software to get a fully automated collateral score (CS) (14), that provides an objective quantification that is much more reproducible by other researchers.

The aim of the current study was to determine the association of admission BP with the degree of CC using an automated CS and to determine the prognostic value of CC in patients with AIS treated with EVT.

## Materials and methods

### Study population

We conducted a retrospective study of all consecutive AIS patients included in our single-center prospective registry from January 2018 to December 2019. Study inclusion criteria were: (1) age ≥18 years, (2) intracranial anterior circulation LVO (M1, M2, or terminal internal carotid artery (TICA) with or without occlusion of extracranial internal carotid artery) treated with EVT. We excluded patients with either: (1) those with an occluded artery in addition to those mentioned above, (2) presence of intracranial hemorrhage on baseline CT, (3) previous anterior circulation territorial infarct, or (4) extreme artifacts on baseline CT or technical issues precluding image analysis.

The following variables were recorded for all of the patients: demographic data: age and sex; vascular risk factors: previous stroke, ischemic stroke or transient ischemic attack within the week before the index stroke, arterial hypertension, diabetes, hypercholesterolemia; drug treatment at admission (statins, antiplatelet, anti-coagulants, antihypertensives, beta-blockers); previous mRS score; clinical data: admission BP, National Institutes of Health Stroke Scale (NIHSS) score at admission; blood test parameters: serum urea and creatinine, hemoglobin, haematocrit, glycaemia, platelet count, haemostasis; logistics and metrics: time from stroke onset or last known well to admission at the Emergency department <6 h, door-to-groin puncture time. Etiologic classification of stroke by the TOAST criteria (15). Admission BP was the first value recorded as part of routine clinical care on admission to the emergency department with a non-invasive blood pressure cuff (GE Dinamap^®^ and GE Critikon^®^ cuff). The time of admission to BP is the same as the time of admission to the emergency department.

### Imaging analysis

CT and single-phase CTA were performed on a Philips Brilliance iCT 256-slice scanner. Intracranial LVO identified in the CTA was recorded. Automated and validated (14, 16) software was used to generate the ASPECTS (Alberta Stroke Program Early CT Score) and Collateral Score (CS) (Brainomix Ltd., Oxford, UK). Processed images had a maximum slice thickness of 1 mm.

**Figure 1:**
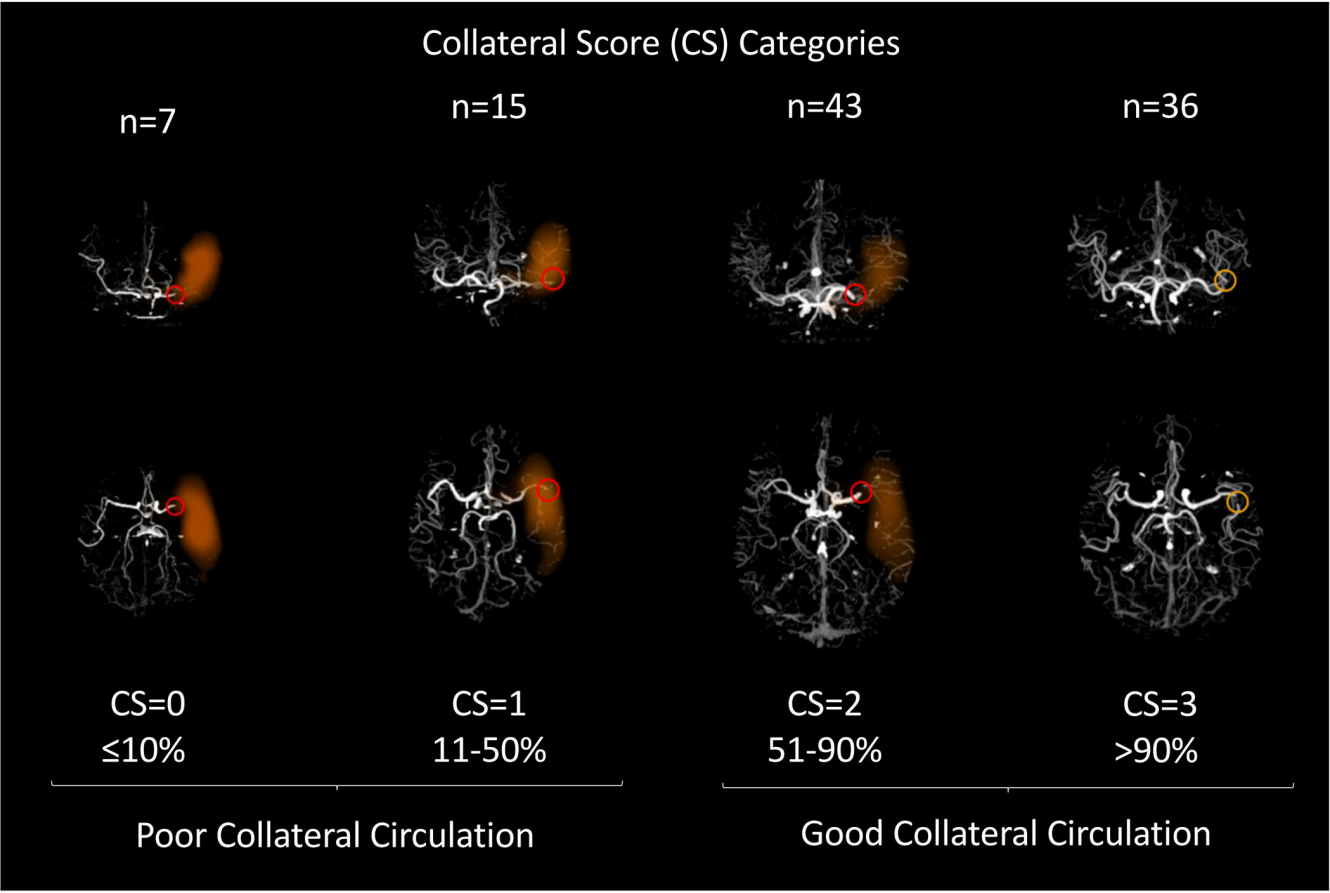
Selected images of our patients processed with e-Stroke (Brainomix Ltd.) are shown as illustrative examples for each CS: 0, collateral filling of ≤10% of the occluded MCA territory; 1, 11–50%; 2, 51–90%; 3, >90%. The first row shows the number of patients in each group in our study. The orange area indicates the area without collateral circulation, the red circle indicates the location of the arterial occlusion.

The CS described by Tan et al. (13) for the evaluation of CC comprises a score ranging from 0 to 3: 0, no collateral filling; 1, ≤50% but >0 of the occluded MCA territory; 2, >50% but <100% of the occluded MCA; 3, 100% collateral supply to the occluded MCA territory. Because the software gives an exact quantitative value, it is very unlikely to get 0% or 100% accurate. Thus, the automatically obtained CS in our study was graded as follows: 0, collateral filling of ≤10% of the occluded MCA territory; 1, 11–50%; 2, 51–90%; 3, >90%. When dichotomized, we considered that CS was good for a CS of 2 or 3, and poor for a CS of 0 or 1 (see Figure 1). The acquisition phase of the CTA is given by the software (early arterial, peak arterial, equilibrium, and peak venous and late venous). The acquisition phase of the study may influence the CS, an earlier acquisition phase can underestimate the CS.

The automated analysis was performed after patient care and for research purposes only; it was not used for the selection of patients to be treated with EVT.

EVT success was measured using the thrombolysis in cerebral infarction (TICI) score.

### Outcomes

The primary end-point was to determine whether the admission BP values may be related to the degree of CC as classified by the automated CS. The secondary outcome was to determine the influence of automated CS on functional outcome at 3 months. At 90 days, a stroke neurologist evaluated the functional outcome with the mRS. We defined favorable functional outcomes as mRS 0–2 at 3 months for patients with a previous mRS score of 0–2. For patients with a previous mRS score of 3, the outcome was favorable when the score at discharge was 3.

### Statistical analysis

Continuous variables were reported as means and standard deviations or medians and interquartile range (IQR) if they were not-normally distributed, as tested by the Shapiro–Wilk normality test. Categorical variables were expressed as counts and percentages.

We classified the study population according to the four different categories of CS. Bivariate analyses were performed? between these groups using ANOVA or the Kruskal–Wallis test (when a non-parametric test was required) for continuous variables, and the χ2 test for categorical variables. Thereafter, we performed a multivariable ordinal logistic regression analysis to predict CS categories in our population. From an initial model including all the variables with *p* < 0.1 in the bivariate analysis, we performed a stepwise backward regression modeling to select variables independently associated with CS. The final model was adjusted for the CTA acquisition phase.

We performed a shift analysis using the unpaired Kruskal–Wallis test to demonstrate differences in the mRS score distribution at 3-months of follow-up between patients with different CS. In the shift analysis, we calculated the common odds ratio (OR) of worsening by 1 point on the mRS according to the CS (unadjusted and adjusted for age) using ordinal logistic regression. We performed a subgroup analysis considering only those patients who reached successful reperfusion according to the TICI 2b/2c/3.

Finally, to evaluate the functional outcomes, we dichotomized the study population as presenting favorable (mRS = 0–2) or unfavorable functional outcomes (mRS = 3–6). We performed bivariate analysis and multivariable ordinal logistic regression analysis as indicated in the previous paragraph and multivariable logistic regression analysis after dichotomising CS. We considered the final TICI as an intermediate outcome in the pathway to functional outcomes at 3 months, we have excluded it from the final multivariable models.

Statistical significance for all the analyses was set at 0.05 (two-sided). All the analyses were performed using Stata v.15 (Texas, USA).

## Results

### Study population

Over a period of two years, we treated 130 AIS patients with EVT in our center. We included 101 patients (see Flowchart in Supplementary Figure I) with a mean age of 72.1 (SD = 13.1), 57 (56.4%) of them were women, and 98 (97%) had a previous mRS = 0–2. We treated 41 patients (40.6%) with recombinant tissue-type plasminogen activator (r-tPA). We found no significant differences in the proportion of patients treated with r-tPA between CS subgroups (*p* = 0.081). Characteristics of the population are detailed in Table 1.

**Table 1:** Bivariate analyses of predictors of collaterals classified by CS categories.

	**All (*n* = 101)**	**CS = 0 (*n* = 7)**	**CS = 1 (*n* = 15)**	**CS = 2 (*n* = 43)**	**CS = 3 (*n* = 36)**	***p***
Age, mean (SD)	72.1 (13.1)	74.9 (8.4)	77.7 (10.6)	71.8 (13.3)	69.5 (14.2)	0.217
Sex (woman), *n (%)*	57 (56.4)	4 (57.1)	7 (46.7)	24 (55.8)	22 (61.1)	0.823
Baseline NIHSS, median (IQR)	16 (9–20)	20 (17–22)	21 (18–23)	13 (8 (20)	11 (9–19)	<0.001
Stroke onset–to–door <6h, *n* (%)	76 (75.25)	7 (100)	12 (80)	33 (76.7)	24 (66.7)	0.266
Door–to–groin puncture time, median (IQR)	62 (55–76)	51 (48–59)	62 (55–76)	65 (57–79)	62 (52–78)	0.108
ASPECTS, median (IQR)	9 (8–10)	8 (8–9)	8 (7–10)	9 (9–10)	9 (9–10)	0.098
M1 occlusion, *n* (%)	52 (51.5)	4 (57.1)	10 (66.7)	22 (51.1)	16 (44.4)	0.534
M2 occlusion, *n* (%)	28 (27.7)	0 (0.0)	3 (20.0)	15 (34.9)	10 (27.8)	0.237
TICA occlusion, *n* (%)	12 (11.9)	3 (42.9)	1 (6.7)	2 (4.7)	6 (16.7)	0.021
Tandem occlusion, *n* (%)	16 (15.9)	1 (14.3)	3 (20.0)	6 (14.0)	6 (16.7)	0.952
Cardioembolic, *n (%)*	49 (48.5)	4 (57.1)	7 (46.7)	22 (51.1)	16 (44.4)	0.899
Large–artery atheromatosis, *n* (%)	13 (12.9)	0 (0.0)	3 (20.0)	5 (11.6)	5 (13.9)	0.614
Hypertension*, *n* (%)	70 (70.7)	7 (100)	9 (60.0)	32 (76.2)	22 (62.9)	0.146
Diabetes**, *n* (%)	19 (19.3)	2 (28.6)	4 (26.7)	6 (14.7)	7 (20.0)	0.685
Dyslipidemia**, *n* (%)	47 (48.0)	5 (71.4)	7 (50.0)	22 (52.3)	13 (37.1)	0.316
Statins*, *n* (%)						
No statin Low intensity Moderate intensity High intensity	68 (68.7) 4 (4.0) 18 (18.2) 9 (9.1)	4 (57.1) 1 (14.3) 1 (14.3) 1 (14.3)	8 (53.3) 0 (0.0) 5 (33.3) 2 (13.3)	30 (71.4) 2 (4.8) 8 (19.1) 2 (4.8)	26 (74.3) 1 (2.9) 4 (11.4) 4 (11.4)	0.546
Antihypertensive*, *n* (%)						
No drugs 1 drug 2 or more drugs	37 (37.4) 15 (15.1) 47 (47.5)	0 (0.0) 2 (28.6) 5 (71.4)	6 (40.0) 2 (13.3) 7 (46.7)	11 (26.2) 6 (14.3) 25 (59.5)	20 (57.1) 5 (14.3) 10 (25.6)	0.037
Previous stroke*, *n (%)*	6 (6.1)	1 (14.3)	0 (0.0)	1 (2.4)	4 (11.43)	0.206
Stroke or TIA previous week*, *n* (%)	8 (8.1)	0 (0.0)	0 (0.0)	4 (9.52)	4 (11.43)	0.461
Hemoglobin*, median (IQR) g/L	134 (121–144)	138 (124–153)	136 (128–140)	133 (120–147)	130 (115–141)	0.438
Hematocrit***, median (IQR) L/L	0.4 (0.37–0.42)	0.4 (0.38–0.46)	0.4 (0.39–0.41)	0.4 (0.36–0.43)	0.4 (0.35–0.42)	0.545
Glycaemia****, median (IQR) mg/100mL	121 (103–146)	145 (132–161)	125 (106–153)	115 (102–134)	120 (99–131)	0.1361
Creatinine*, median (IQR) micromol	77 (67–91)	77 (75–112)	83 (60–108)	84 (72–96)	70 (63–82)	0.048
Admission systolic blood pressure, mean (SD)	155 (26)	154 (30)	169 (29)	160 (25)	143 (22)	0.003
Admission mean blood pressure, mean (SD)	105 (15)	105 (12)	111 (17)	109 (14)	99 (14)	0.020
Admission diastolic blood pressure, mean (SD)	81 (13)	81 (5)	81 (13)	83 (12)	78 (13)	0.252

Missing information for ^*^2, ^**^ 3, ^***^ 4 and ^****^ 24 patients.

CS (Collateral Score: 0, collateral filling of ≤10% of the occluded middle cerebral artery (MCA) territory; 1, 11–50%; 2, 51–90%; 3, >90%), NIHSS (National Institutes of Health Stroke Scale), Stroke onset–to–door < 6h (time from stroke onset or last know well to admission at the Emergency department < 6h), Door–to–groin puncture time is expressed in minutes, ASPECTS (Alberta Stroke Program Early CT Score), TICA (Terminal Internal Carotid Artery), TIA (Transient Ischemic Attack), U/C ratio (Urea mmol /Creatinine mmol ratio).

### Primary outcome

We classified patients into groups based on their category in the CS: 7 patients (6.9%) were classified as CS = 0, 15 (14.9%) as CS = 1, 43 (42.6%) as CS = 2 and 36 (35.6%) as CS = 3. As shown in Table 1, we found no significant differences in age, sex, or stroke etiology among the four groups. In the bivariate analysis, we found that patients with a higher CS score had a lower baseline NIHSS score, were taking fewer antihypertensive drugs prior to admission, had lower serum creatinine levels, and had lower admission systolic and mean BP. Patients classified as CS = 0 had a non-significant (*p* = 0.263) lower admission systolic and median BP than the CS = 1 group. Among patients with a CS = 0, there was a higher percentage of TICA occlusion. Because mean BP is calculated from systolic BP and diastolic BP, we conducted two multivariable ordinal logistic regression analyses, model 1 with systolic BP and model 2 with mean BP (see details in Table 2). In model 1, higher systolic BP [aOR for 10 mmHg increase 0.79 (95% CI 0.68–0.92)] and higher baseline NIHSS [aOR 0.90 (95% CI, 0.84–0.96)] were independent variables associated with a lower CS. Similarly, in model 2, higher mean BP [aOR for 10 mmHg increase 0.70 (95% CI 0.54–0.90)] and higher baseline NIHSS [aOR 0.90 (95% CI, 0.83–0.95)] were associated with a lower category of CS.

**Table 2:** Results of the multivariable ordinal logistic regression analysis of predictors of good collaterals classified by collateral score (CS) categories.

	**aOR**	**95% CI**	***p***
*Model 1*			
Systolic blood pressure (x1 mmHg increase) (x10 mmHg increase)	0.98 0.79	0.96–0.99 0.68–0.92	0.003
Baseline NIHSS	0.90	0.84–0.96	0.002
*Model 2*			
Mean blood pressure (x1 mmHg increase) (x10 mmHg increase)	0.96 0.70	0.94–0.99 0.54–0.90	0.006
Baseline NIHSS	0.90	0.83–0.95	0.001

NIHSS (National Institutes of Health Stroke Scale).

Adjusted for Computed Tomography Angiography acquisition phase. OR for worsening in one CS category.

### Secondary outcome

**Figure 2:**
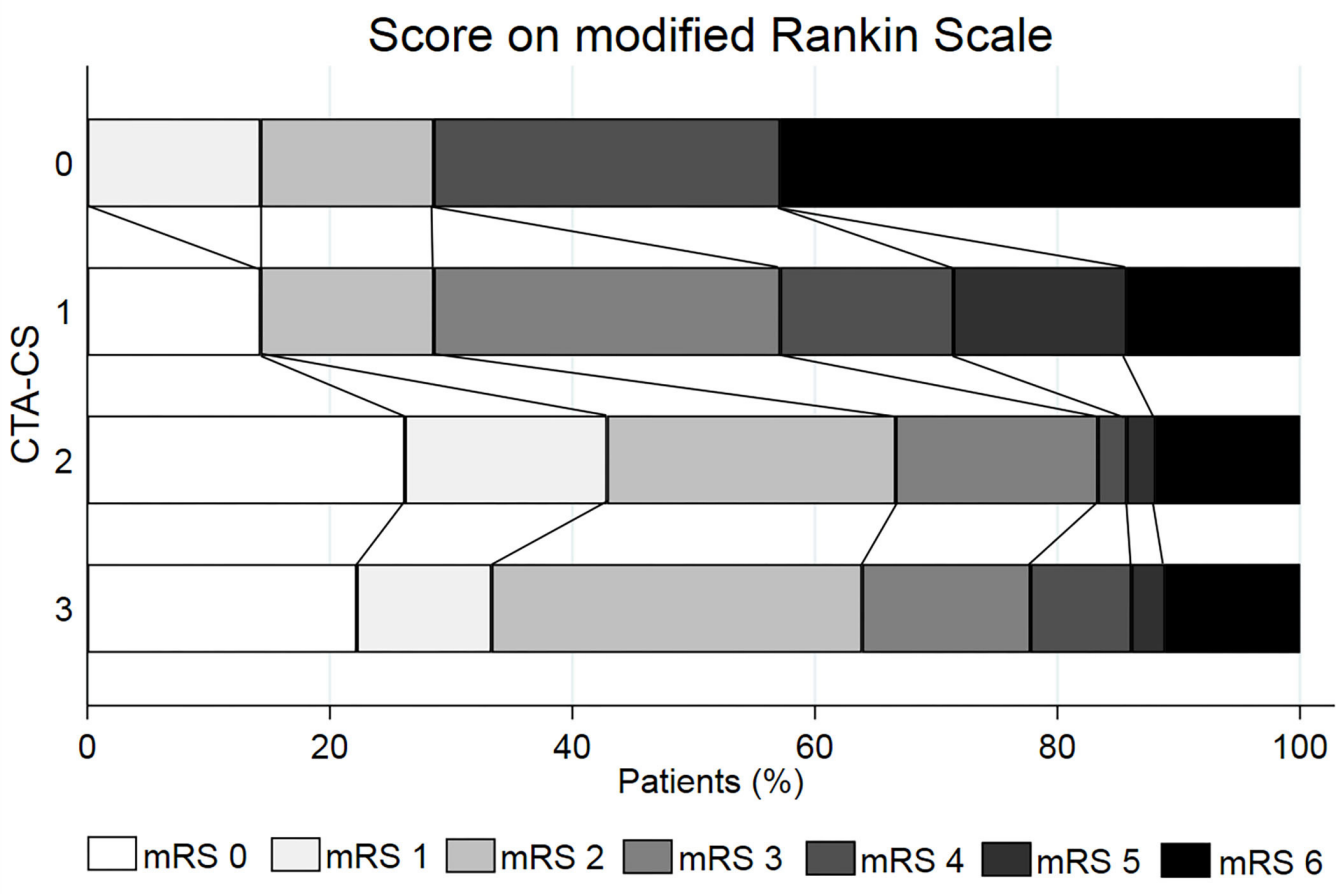
Distribution of the mRS score at 3 months according to the baseline automated determined Collateral Score (CTA-CS).

The shift analysis showed a median of mRS at 3 months of 4 (IQR 2–6) in the CS = 0 group, 3 (IQR 2–5) in the CS = 1 group, 2 (IQR 0–3) in the CS = 2 group, and 2 (IQR 1–3) in the CS = 3 group (*p* = 0.024) (see Figure 2). The OR of improving 1 point on the 3-month mRS was 1.63 (95% CI, 1.10–2.44) favoring a better CS (*p* = 0.016). After adjusting by age, the common odds ratio was 1.58 (95% CI, 1.05–2.39, *p* = 0.027). When considering only patients with successful recanalization (TICI 2b/2c/3) the CS category was also associated with the probability of achieving a better mRS score at three months with a common OR of 1.59 (95% CI, 1.01–2.49, *p* = 0.044). CS was no longer associated with mRS score when adjusted by baseline NIHSS.

At 3 months of follow-up 57 (57.6%) patients presented a favorable outcome. A better CS category was related to a better clinical outcome at 3 months in the bivariate analysis (*p* = 0.026). However, the significance was lost in the multivariable logistic regression analysis (Models 1 and 2). Details of bivariate analyses are shown in Supplementary Table I and of the multivariable logistic regression analyses are shown in Table 3.

**Table 3:** Results of the multivariable logistic regression analysis of predictors of good functional outcome at 90 days of follow–up.

	**OR**	**95% CI**	***p***
*Model 1*			
Collateral Score (CS)	1.39	0.80–2.42	0.240
Baseline NIHSS	0.90	0.83–0.97	0.009
Previous mRS	0.50	0.30–0.80	0.005
*Model 2*			
Good vs. poor Collaterals	2.64	0.78–9.00–	0.120
Baseline NIHSS	091	0.83–0.99	0.021
Previous mRS	0.50	0.31–0.81	0.005

Poor Functional Outcome was defined as mRS = 3–6, mRS (modified Rankin Scale), Good Collaterals included CS = 2–3, Poor Collaterals included CS = 0–1, TICI score (thrombolysis in cerebral infarction score).

## Discussion

In patients with AIS secondary to LVO who were treated with EVT, we found that a higher BP at admission was associated with poorer automated CS on baseline CTA. In addition, a better automated CS was associated with a better functional outcome at 3 months of follow-up.

Prior studies reported that higher admission BP is associated with a higher final infarct volume, a lower probability of successful reperfusion, and with a lower likelihood of favorable clinical outcomes in AIS patients treated with EVT (6, 17). However, the relationship between baseline BP and CC has been barely described. One study of AIS patients treated with intravenous thrombolytic therapy (with or without LVO) found that moderately elevated systolic BP was associated with good CC but with an inverse association of systolic BP and 3-month clinical outcome (8); another study found that lower systolic BP was associated with good CC but in patients with ICA occlusion during the first 3 days from stroke onset (9). In this regard, our study adds relevant information confirming that both systolic BP and mean BP at admission are inversely associated with the CS.

Pathophysiological knowledge suggests that the direction of this independent relationship is that poor CC leads to increased BP. LVO causes a drop in perfusion pressure, which leads to recruitment and vasodilatation of the leptomeningeal collaterals and, due to impaired cerebral autoregulation, the direction and amount of blood flow through CC rely on systemic perfusion, which can be estimated by BP (18). Until successful reperfusion, the survival of hypoxemic brain tissue depends on CC flow, and for this reason guidelines do not recommend lowering BP unless it is extremely high (19). In this regard, in the bivariate analysis we found a relationship between a history of taking a greater number of antihypertensive drugs before admission and a worse CS in baseline CTA. It should be noted that in the CS = 0 subgroup (which had a lower BP than the others although not significant probably because of its small size) all patients were taking antihypertensive drugs.

We also found an independent association in the multivariable analysis between more severe strokes (as defined by a higher NIHSS score) and worse CS. Probably the direction of this relationship is probably poorer CS implying lower blood flow to the penumbra area (5), a larger ischemic core (20), and thus greater clinical severity.

Our study confirms that a better CC status is associated with a better clinical outcome following EVT. A neuroimaging meta-analysis of pivotal clinical trials of EVT, reported that a better CC status was associated with a better NIHSS within the first 24 h and a better clinical outcomes at 3 months (21). Our study confirms that CS is an independent prognostic factor after adjusting by age and when considering only those achieving a successful recanalization. When adjusting for NIHSS, CS lost significance as a prognostic factor, but we believe this is due to the relationship we have already discussed: the worse the CC, the higher the NIHSS.

Assessment of CS can be time-consuming, subjective, and the challenge for the manual scoring system is the low agreement between evaluators, making patient characterization less consistent. Several studies have shown that fully automated or semi-automated methods are a quantitative, reliable, time-saving, and user-independent measure of CS on baseline CTA (12, 14, 22). Semiautomated CS evaluation has been associated with clinical outcome previously in AIS patients treated with EVT (22), however, to the best of our knowledge the relationship between admission BP and automatically measured CS had not been previously described.

Our study has some limitations. Patients treated with EVT may have a selection bias by causing the number of patients with good CS to be higher in this study than in the general population. Collateral circulation assessed by using perfusion CT or multiphase CTA was significantly better than those assessed by CTA, however, the results obtained by CTA are much more reproducible due to their availability. Neither BP nor CC are static, and in this study, we have only one measurement of each variable, without being able to analyse what happens in the rest of the acute process.

In conclusion, in patients with an AIS due to anterior LVO, admission BP was independently and inversely associated with automated CS on baseline CTA. Moreover, automated CS was associated with clinical outcomes at 3 months. Prospective data and clinical trials to determine the best management of BP and other modifiable factors to maintain optimal CC peri-EVT are needed.

## Data availability statement

The data that support the findings of this study are available from the corresponding author upon reasonable request.

## Ethics statement

The Ethics Committee of Hospital de la Santa Creu i Sant Pau reviewed and approved this study (IIBSP-COL-2019-120). Written informed consent for participation was not required for this study in accordance with the national legislation and the institutional requirements.

## Author contributions

DG-A and JM-F designed and conceptualized the study. DG-A and PC-R major role in the acquisition of data. DG-A, JM-F, PC-R, and RD-M analyzed and interpreted the data. DG-A wrote the first draft of the manuscript. DG-A, PC-R, RD-M, EG, AM-D, LP-S, MG-J, MA, AR-P, and JM-F reviewed and edited the manuscript and approved the final version of the manuscript.

## Funding

This work was supported by Redes de Investigación Con Objetivos de Resultados en Salud (RICORS) RD21/0006/0006, Instituto de Salud Carlos III, Ministry of Science and Innovation (Government of Spain). DG-A (CM18/00065) and MG-J (CM20/00056) received a Río Hortega Research grant from the Instituto de Salud Carlos III, Ministry of Science and Innovation (Government of Spain). Collateral Circulation (collaterome) in Acute Ischemic Stroke with Large Vessel Occlusion: A Study of Clinical, Radiological, Plasma and Genetic Factors, Proyecto de investigación en salut (PI19/00859), Instituto de Salud Carlos III, Ministry of Science and Innovation (Government of Spain). This study has been co-funded by the European Union through the Fondo Europeo de Desarrollo Regional (FEDER).

## Conflict of interest

Brainomix has provided its software free of charge to the Stroke Unit of the Hospital de la Santa Creu i Sant Pau, in accordance with the regulations of the Biomedical Research Institute Sant Pau and for research purposes only. The authors declare that the research was conducted in the absence of any commercial or financial relationships that could be construed as a potential conflict of interest.

## Publisher's note

All claims expressed in this article are solely those of the authors and do not necessarily represent those of their affiliated organizations, or those of the publisher, the editors and the reviewers. Any product that may be evaluated in this article, or claim that may be made by its manufacturer, is not guaranteed or endorsed by the publisher.
