# Supplementary material for: Automated scoring of collaterals, blood pressure, and clinical outcome after endovascular treatment in patients with acute ischemic stroke and large-vessel occlusion
Source: Front Neurol. 2022 Aug 9;13:944779. doi: 10.3389/fneur.2022.944779 (PMC9397141; doi:10.3389/fneur.2022.944779)
Supplement: Supplementary file 1 [file Data_Sheet_1.pdf]

Supplementary Material

Supplementary Figure I. Flowchart

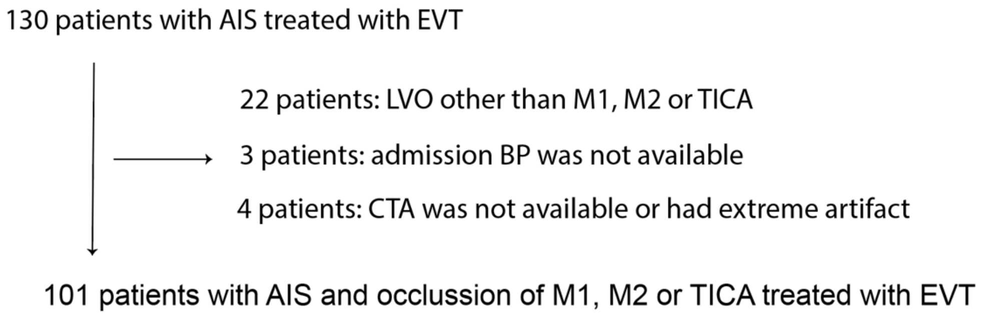

AIS (acute ischemic stroke), EVT (endovascular treatment), LVO (large vessel occlusion), M1 (M1 segment of middle cerebral artery (MCA)), M2 (M2 segment of MCA), TICA (Terminal Internal Carotid Artery), BP (blood pressure), CTA (CT angiography.

Supplementary Table I. Bivariate Analyses of Predictors of Functional Out-come at 90 Days of Follow-Up

	Good functional outcome, mRS=0-2 (n=57)	Poor functional outcome, mRS=3-6 (n=42)	p
Age, mean (SD)	74.6 (11.9)	70.4 (13.9)	0.111
Sex (woman), n (%)	33 (57.9)	23 (54.8)	0.756
Previous mRS, median (IQR)	0 (0-1)	1 (0-2)	0.005
Baseline NIHSS, median (IQR)	11 (8-18)	19 (14-21)	<0.001
ASPECTS, median (IQR)	9 (8-10)	9 (8-10)	0.846
M1 occlusion, n (%)	30 (52.6)	21 (50.0)	0.796
M2 occlusion, n (%)	16 (28.1)	12 (28.6)	0.956
TICA occlusion, n (%)	5 (8.8)	7 (16.7)	0.234
Tandem occlusion, n (%)	9 (15.8)	6 (14.3)	0.837
Cardioembolic, n (%)	26 (45.6)	22 (52.4)	0.506
Atherothrombotic, n (%)	8 (14.0)	4 (9.5)	0.497
Hypertension*, n (%)	35 (63.6)	34 (80.9)	0.062
Diabetes**, n (%)	9 (16.4)	10 (24.4)	0.329
Dyslipidemia**, n (%)	22 (40.7)	24 (57.1)	0.111
Collateral Score (CS), n (%)			
0	2 (3.5)	5 (11.9)	
1	4 (7.0)	10 (23.8)	0.026
2	28 (49.1)	14 (33.3)	
3	23 (40.4)	13 (31.0)	

Good collateral status (vs. poor), n (%)	51 (89.5)	27 (64.3)	0.002
r-tPA	26 (45.6)	13 (31.0)	0.140
Final TICI, median (IQR)	3 (2c-3)	2b (2b-3)	<0.001
Admission systolic blood pressure, mean (SD)	151 (24)	158 (29)	0.187
Admission mean blood pressure, mean (SD)	104 (14)	107 (17)	0.324
Admission diastolic blood pressure, mean (SD)	80 (12)	81 (14)	0.681

*Missing information for 4 patients

**Missing information for 5 patients

mRS (modified Rankin Scale), ASPECTS (Alberta Stroke Program Early CT Score), TICA (Terminal Internal Carotid Artery), CS (Collateral Score: 0, ≤10% of the occluded MCA territory; 1, ≤50% but > 10% of the occluded MCA territory; 2, > 50% but ≤ 90% of the occluded MCA; 3, >90% collateral supply of the occluded MCA territory), Good Collaterals included CS=2-3, Poor Collaterals included CS=0-1, r-tPA (patients treated with recombinant tissue-type plasminogen activator), TICI scale (thrombolysis in cerebral infarction scale: 0, 1, 2a, 2b, 2c, 3).
